# HER2 Therapies in Non-Small Cell Lung Cancer (NSCLC)

**DOI:** 10.3390/ijms27114910

**Published:** 2026-05-29

**Authors:** Fedor Wadi Richani Meinhardt, Mijail I. Zambrano Iglesias, María P. Fernández Gómez, Jesús F. Saltaren Fonseca, Atif Hussein, Luis E. Raez

**Affiliations:** 1 Memorial Cancer Institute, Pembroke Pines, FL 33027, USA; mzambranoiglesias@mhs.net (M.I.Z.I.); mfernandezgomez@mhs.net (M.P.F.G.); ahussein@mhs.net (A.H.); lraez@mhs.net (L.E.R.); 2 Independent Researcher, Rio de Janeiro 22210-030, Brazil; jesus.saltaren@gmail.com; 3 Institute for Human Health and Disease Intervention, Florida Atlantic University (FAU), Jupiter, FL 33458, USA

**Keywords:** HER2, ERBB2, non-small cell lung cancer, exon 20 insertion, antibody–drug conjugate, trastuzumab deruxtecan, zongertinib, sevabertinib, immunohistochemistry, next-generation sequencing

## Abstract

This review discusses the role of human epidermal growth factor receptor 2 (HER2/*ERBB2*) as a key oncogenic driver in non-small cell lung cancer (NSCLC), including exon 20 activating mutations, gene amplification, and protein overexpression. These forms differ in their biological effects and predictive value, but HER2 mutations, especially exon 20 insertions, are the primary oncogenic mechanism. Regarding diagnosis, Next-Generation Sequencing (NGS) is used to identify mutations, whereas Immunohistochemistry (IHC) and in situ hybridization are used to assess HER2 expression. Concerning treatment, in advanced HER2-positive, Non-Squamous NSCLC tumors, the first-line treatment is Platinum-based + Pemetrexed chemotherapy, with or without immunotherapy, because no HER2-targeted antibody therapy has yet been approved for initial treatment. After progression, HER2-targeted antibody-drug conjugates like Trastuzumab-Deruxtecan and Ado Trastuzumab-Emtansine may offer patients clinical benefits. New HER2-selective tyrosine kinase inhibitors, such as zongertinib and sevabertinib, have shown promising results, including patients previously treated with antibody–drug conjugates (ADCs). Recent advances, including next-generation ADCs such as SHR-A1811 and A166, and bispecific antibodies, such as zenocutuzumab for NRG1 fusion–positive disease, which are also expanding treatment options. Overall, advances in diagnostics and new targeted therapies are changing how HER2-altered NSCLC is treated and are helping to make care more personalized.

## 1. Introduction

**Figure 1:**
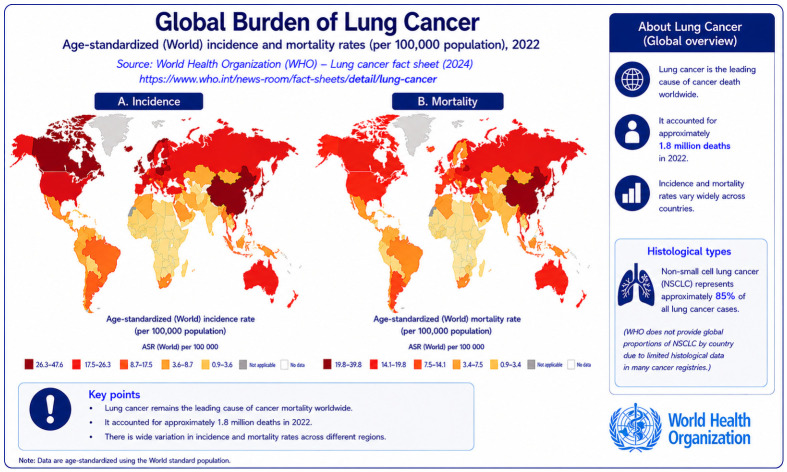
Global age-standardized incidence (**A**) and mortality (**B**) rates of lung cancer per 100,000 population in 2022, highlighting regional variability and the substantial global burden of NSCLC, which accounts for approximately 85% of all lung cancer cases and remains the leading cause of cancer-related death worldwide. Figure created using data from the WHO Lung Cancer Fact Sheet [1] and the Global Cancer Observatory: Cancer Today database [2].

Lung cancer remains the leading cause of cancer-related mortality worldwide, accounting for approximately 1.8 million deaths annually. Non-small cell lung cancer (NSCLC) represents approximately 85% of all lung cancer cases [1,2] (Figure 1). At the molecular level, NSCLC is characterized by alterations in key oncogenic signaling pathways, particularly those involving receptor tyrosine kinases such as the human epidermal growth factor receptor (HER/ErbB) family.

The Human Epidermal Growth Factor Receptor (HERs), also known as Erythroblastic Leukemia Oncogene B (ErbB) family [3], encoded by the ErbB oncogenes, comprises four structurally related transmembrane receptor tyrosine kinases, including HER1/EGFR (ErbB1), HER2 (ErbB2/Neu), HER3 (ErbB3), and HER4 (or ErbB4), each defined by unique ligand-binding characteristics, which regulate key cellular processes including proliferation, differentiation, and survival [4,5]. This pathway became well known because HER2 amplification was proven to be oncogenic and responsible for breast cancer, for more than 20 years several new agents have been developed targeting this gene. More recently, members of the c-ErbB oncogene family, particularly EGFR and ERBB2 (HER2), have been extensively implicated in the pathogenesis of non-small cell lung cancer (NSCLC), where their aberrant activation and dysregulated signaling promotes uncontrolled cellular proliferation and inhibits apoptosis [6,7]. These effects are mediated through key downstream signaling cascades, including the PI3K/AKT/mTOR and RAS/RAF/MEK/ERK pathways, ultimately, driving tumor initiation and progression [7,8,9,10].

### 1.1. Molecular Mechanisms of HER2 in NSCLC

**Figure 2:**
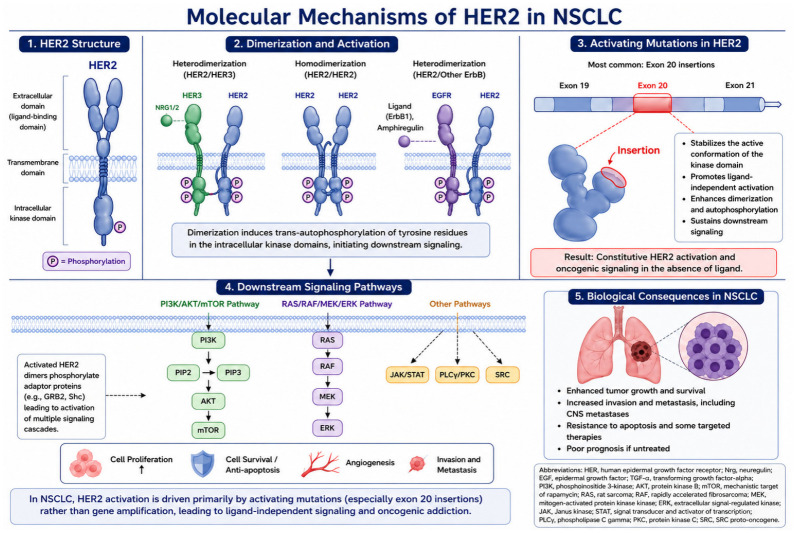
Schematic representation of HER2 activation and downstream signaling pathways involved in NSCLC tumorigenesis, including PI3K/AKT/mTOR and MAPK signaling cascades. Figure created by the authors based on information summarized from Refs. [5,11,17,20,21].

Ligand binding induces homo- or heterodimerization of ErbB receptors, leading to the activation of their intracellular kinase domains and downstream mitogenic signaling pathways, including the RAS–MAPK and PI3K–AKT cascades (Figure 2). While HER1 (EGFR) binds ligands such as TGF-α and amphiregulin, no direct ligand has been identified for HER2; however, neuregulin isoforms function as ligands for HER3 and HER4 [9,11].

HER2 is encoded by the ERBB2 gene, located on chromosome 17q12, producing a ~185 kilodaltons (kDa) receptor with extracellular, transmembrane, and intracellular kinase domains [12]. Structurally, similar to other ErbB receptors, HER2 contains an amino-terminal ligand binding region, a single pass transmembrane helix, and a Carboxy-terminal tyrosine kinase domain (TKD) with autophosphorylation sites for downstream signaling effector recruitment [13,14,15]. However, unlike EGFR and HER3/4, HER2 lacks a known ligand and is activated through dimerization with ligand-bound receptors, particularly HER3, enhancing signal transduction efficiency via MAPK and PI3K/AKT pathways. This unique receptor configuration underpins its potent mitogenic potential when dysregulated in cancer [5,11,14].

HER2 mutations occur in approximately 2–4% of NSCLC as activating mutations, gene amplifications in 2–5%, and protein overexpression has been described around 13–20% [16,17]. These differences reflect distinct biological mechanisms rather than conflicting data, as HER2 overexpression in NSCLC is not strongly correlated with gene mutations or amplification, unlike in other tumor types. These alterations are more frequently observed in adenocarcinomas, women, and never-smokers, and are associated with aggressive clinical behavior and an increased propensity for central nervous system metastases [9,16,17,18,19]. In NSCLC, HER2 alterations are most commonly activating mutations, particularly exon 20 insertions, which enhance kinase activity and lead to persistent activation of downstream signaling pathways such as PI3K/AKT and MAPK, independent of ligand stimulation [5,20,21] (Figure 2).

The oncogenic role of HER2 is highly context-dependent and varies significantly across malignancies, reflecting distinct mechanisms of activation and therapeutic susceptibility. In breast cancer, HER2-driven disease is primarily characterized by ERBB2 gene amplification leading to protein overexpression, which promotes constitutive receptor homodimerization, strong oncogene dependence, and high sensitivity to monoclonal antibodies such as trastuzumab and pertuzumab [22,23,24,25,26]. A similar pattern is observed in gastric and gastroesophageal junction cancers, where amplification and overexpression predominate, though with greater spatial and temporal tumor heterogeneity that can limit therapeutic response [12,27,28,29,30]. In contrast, HER2 alterations in NSCLC are most commonly activating mutations rather than amplification or overexpression, and these alterations occur with minimal overlap between alteration subtypes [5,19,21,31]. These intertumor differences underscore the critical importance of alteration-specific biological context when interpreting HER2 status and selecting targeted therapies [12,21,26,28,32,33].

Collectively, these features define a distinct HER2-driven biological context in NSCLC compared to other malignancies. In this setting, tumorigenesis is primarily driven by activating kinase domain mutations, most commonly exon 20 insertions, rather than gene amplification, leading to constitutive, ligand-independent downstream signaling. Unlike HER2-amplified tumors, HER2-mutant NSCLC does not consistently exhibit high levels of receptor overexpression and may demonstrate variable degrees of oncogene dependency. Because the primary oncogenic alteration resides within the intracellular kinase domain rather than the extracellular receptor surface, this may limit the effectiveness of extracellular-targeting monoclonal antibody strategies in this setting. This mechanistic context may partially explain the superior activity described with ADCs, which can exploit HER2 surface expression at sub-amplification levels to deliver cytotoxic payloads, including through a bystander effect, and with selective tyrosine kinase inhibitors that directly target the mutant kinase domain. These differences highlight the importance of considering tumor-specific biological context when interpreting HER2 alterations and selecting targeted therapies [5,19,21,31,32,34].

### 1.2. Principal Molecular Forms of HER2 Alterations

I. HER2 gene amplification (2–5%). Increased copy number of the ErbB2 locus, resulting in higher membrane receptor density [8,35]. High-level amplification reliably predicts HER2 overexpression in tumor cells and has been noted both as a primary event and as an acquired resistance mechanism to targeted therapies, particularly EGFR tyrosine kinase inhibitors (TKIs) [36]. These genetic aberrations are actionable in breast cancer de novo and not commonly in lung cancer until there is disease progression in EGFR tumors, as we mentioned before.
II. Somatic mutations in the ErbB2 coding sequence (1–4% approximately). Those activating mutations confer constitutive tyrosine kinase activity [35]. These are the main targets for now in lung cancer patients, where several agents have been approved. In lung cancer, HER2 mutations predominantly affect the tyrosine kinase domain, according to the phase I/II SOHO-1 Trial BAY 2927088 the HER2 exon 20 insertion mutation Y772_A775dup (YVMA, historically00lso annotated A775_G776insYVMA) represents up to 75% of HER2-mutant NSCLC [19,37,38]. This alteration leads to constitutive kinase activation, increased ATP affinity, persistent downstream signaling, and reduced sensitivity to early inhibitors. These mutations cause constitutive activation of the receptor and resistance to traditional therapies to earlier-generation EGFR/HER2 TKIs [8,19]. Less common extracellular or transmembrane variants may also induce abnormal dimerization [19]. Additional recurrent exon 20 variants include G776delinsVC/LC/VV/IC and occasional extracellular or transmembrane missense alterations at sites such as S310X, which may also activate downstream signaling [8,39].
  Many of the kinase-domain HER2 exon 20 insertions are referred to as PACC mutations (p-loop αC-helix compression mutations) and can be defined as a conformational compression of the αC-helix and ATP-binding pocket remodeling, resulting in constitutive kinase activation, making them resistant to first-generation TKIs. The Structure-based classification of these alterations predicts drug sensitivity more accurately than exon-based grouping [40].
III. Protein overexpression (13–20%). Usually detected by IHC, often correlating with amplification in other tumor types, does not strongly correlate with mutation or amplification status in NSCLC, nor has it been consistently predictive of response to HER2-targeted therapy in clinical trials. Mutations serve as the primary oncogenic drivers and amplification/overexpression have variable oncogenic significance [35]. The Food and Drug Administration (FDA) has approved IHC expression in some cases: Trastuzumab deruxtecan received accelerated approval for patients with unresectable or metastatic HER2-positive or solid tumors who have received prior systemic therapy and have no alternative options.
IV. HER2 (ERBB2) gene fusions are rare but actionable oncogenic drivers in various cancers, including lung, breast, and gastric cancers. These rearrangements, often involving upstream partners, lead to constitutive HER2 signaling, mimicking HER2 amplification. While less common than mutations, they represent a potential target for ADCs like trastuzumab deruxtecan [41].

Even though there is increasing interest in HER2 alterations in NSCLC, the therapeutic landscape has rapidly advanced in recent years with the development of ADCs and next-generation HER2-selective TKIs. For that reason, an updated synthesis of molecular mechanisms, diagnostic approaches, and emerging therapeutic strategies is needed. In this review, we condense the current knowledge on HER2 alterations in NSCLC and provide an overview of established treatments and novel targeted therapies that are currently under clinical investigation. The aim of this review is to provide an updated synthesis of HER2 biology, diagnostic strategies, and evolving therapeutic approaches, with a focus on emerging targeted therapies and future clinical directions.

HER2 expression in NSCLC is commonly evaluated by IHC, which assesses membranous staining intensity in tumor cells using assays such as Ventana 4B5 or HercepTest [42,43]. HER2 expression is scored on a 0–3+ scale, where IHC 3+ indicates strong membranous staining and corresponds to high-level HER2 overexpression. In contrast, IHC 1+ and IHC 2+/FISH-negative tumors are generally considered HER2-low. IHC 2+ represents moderate or incomplete membranous staining and is regarded as equivocal, which may require additional assessment by fluorescence in situ hybridization (FISH) to determine HER2 amplification status [42,43]. Overall, HER2 expression has been reported in approximately 13–20% of NSCLC cases, whereas high-level HER2 overexpression (IHC 3+) is observed in only 2–6% of tumors, predominantly in adenocarcinomas [44]. This distinction is clinically important because low-to-moderate HER2 expression (IHC 1+ or 2+) represents a broader and biologically heterogeneous population, whereas HER2 overexpression is restricted to IHC 3+ tumors with consistently strong membranous staining. Importantly, HER2 protein expression does not consistently correlate with *ERBB2* gene mutations or amplification, highlighting the biological complexity of HER2 alterations in NSCLC.

According to the literature, it has shown limited correlation with gene amplification or mutations in NSCLC and minimal predictive value for early HER2-targeted agents like trastuzumab [45]. However, IHC has emerged as an actionable biomarker, particularly for ADCs. Clinical trials like DESTINY-Lung01/02/03 demonstrated clinical activity (e.g., objective responses in IHC 2+/3+ pretreated tumors) with trastuzumab-deruxtecan (T-DXd), which reinforces HER2 overexpression as a targetable alteration [46,47,48]. NCCN guidelines (2025) recommend HER2 IHC testing (alongside NGS for mutations) in advanced non-squamous NSCLC to identify candidates for this kind of therapies, with FISH/SISH for equivocal IHC 2+ cases. High HER2 expression or gene copy number also carries prognostic significance, including inferior disease-specific survival in female patients [33,44]. Challenges include tumor heterogeneity and the need for NSCLC-adapted scoring [42].

In current clinical practice, HER2 mutation status identified by NGS remains the primary biomarker guiding treatment decisions in NSCLC. HER2 IHC serves as a complementary tool, particularly in identifying candidates for ADCs, and is increasingly integrated with genomic testing to refine patient selection.

## 2. Current Therapies for HER2 in Lung Cancer

HER2 mutations are identified in approximately 2–5% of NSCLC cases, most commonly as exon 20 insertions, and are associated with distinct clinical features and a lack of established first-line targeted therapies [11,18]. These mutations confer a unique biology but do not alter the standard approach to initial systemic therapy in the absence of an approved first-line HER2-targeted agent [11]. In addition, these molecular alterations are commonly found predominantly in females and patients with never smoker status, making more complicated the therapeutic decision considering the tumor mutational burden. For patients with advanced non-squamous NSCLC, including those with HER2 mutations, the National Comprehensive Cancer Network (NCCN, 2025) advises first-line treatment with a platinum-based chemotherapy combination (carboplatin or cisplatin) and pemetrexed, with the option of adding pembrolizumab [5]. Immunotherapy agents such as pembrolizumab, atezolizumab, cemiplimab, durvalumab, and nivolumab/ipilimumab are acceptable options in combination with chemotherapy; however, pembrolizumab plus platinum/pemetrexed remains the most widely used and guideline-endorsed regimen [5,40]. Even though the PD-L1 expression is high in some cases, the objective response to a single immunotherapy agent in patients with HER2 mutations is suboptimal compared to other oncogenic mutations. Maintenance therapy with pemetrexed (with or without pembrolizumab) continues to be standard after induction therapy [5].

KEYNOTE-189 demonstrated that adding pembrolizumab to a platinum–pemetrexed enhances both overall survival and progression-free survival compared with chemotherapy alone in patients with metastatic non-squamous NSCLC [12,40]. While this trial excluded patients with EGFR or ALK alterations, it did not specifically evaluate HER2-mutant populations. Prior analyses of this cohort show that while the benefit exists, the magnitude of the survival advantage may be reduced by the specific structural constraints of the HER2 protein. Real-world and retrospective data suggest that combining immunotherapy with platinum-based chemotherapy in HER2-mutant NSCLC improves progression-free survival (median 8.5 vs. 6.3 months) but not overall survival, compared with chemotherapy alone. Response rates appear similar between chemo-immunotherapy and chemotherapy alone [18,35]. These findings emphasize the critical need for prospective trials integrating targeted agents into the first-line setting to address early chemoresistance. No evidence demonstrates that carboplatin is superior to cisplatin or vice versa when combined with pembrolizumab in HER2-mutant NSCLC; the decision is based on comorbidities and tolerability [5].

After progression, treatment with HER2-specific ADCs, like fam-trastuzumab deruxtecan-nxki (T-DXd), is recommended; however, these agents are not approved for first-line therapy use [5,11]. Both T-DXd and ado-trastuzumab emtansine (T-DM1) deliver potent cytotoxic payloads directly to HER2-expressing tumor cells, achieving meaningful response rates and disease control in previously treated HER2-mutant NSCLC [21,23,33]. T-DXd utilizes a cleavable tetrapeptide-based linker and a topoisomerase I inhibitor payload, which allows for a potent “bystander effect” that targets neighboring tumor cells regardless of their individual HER2 expression levels. NCCN guidelines list T-DXd as the preferred subsequent-line therapy, with T-DM1 as a supported alternative. T-DXd received FDA accelerated approval based on DESTINY-Lung02 [33,49]. Data from DESTINY-Lung01 and DESTINY-Lung02 show objective response rates (ORRs) of 50–58%, median Progression free Survival(mPFS) of 8.7–10 months, and OS of approximately 17.8 months [21,23,33,49,50,51]. Notably, the 5.4 mg/kg dose of T-DXd has emerged as the optimized standard, balancing robust intracranial activity with a more manageable safety profile compared to higher doses tested earlier. T-DM1 demonstrates lower activity, with ORRs of 31–44% and median duration of response of 4–5 months [23,52]. Interstitial lung disease remains the principal safety concern for T-DXd, occurring in 6–14% of cases [23,49,50]. Clinicians must maintain high vigilance and implement early corticosteroid intervention, as grade 5 Interstitial Lung Disease (ILD) events have been reported in the literature. Real-world studies support better outcomes with T-DXd compared with T-DM1 or trastuzumab-based regimens [53].

**Figure 3:**
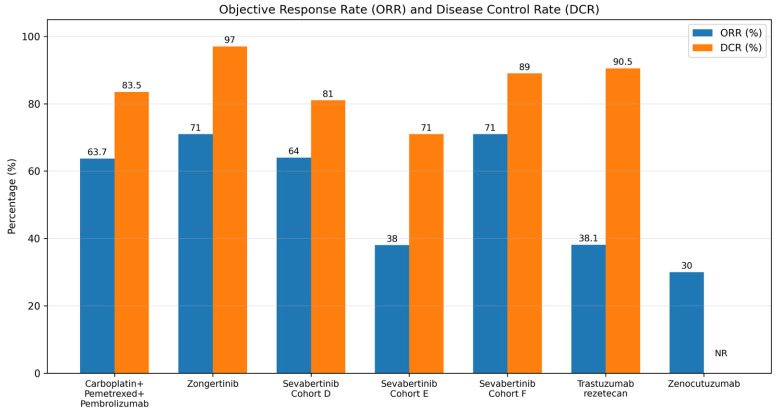
Graphical representation of ORR and DCR summarized in Table 1. Each bar represents results from each study, including cohort-specific outcomes for sevabertinib (BAY 2927088). Results should not be interpreted as direct cross-trial comparisons.

**Figure 4:**
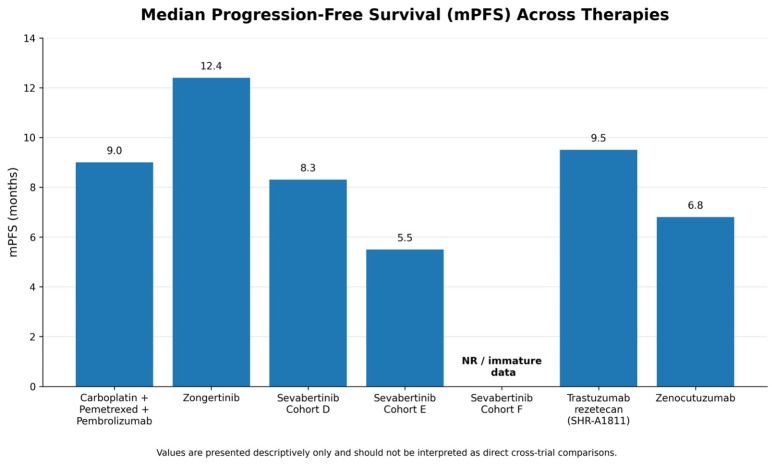
Graphical illustration of median progression-free survival (mPFS, months) across therapies and cohorts summarized in Table 1. **HER2-targeted TKIs show numerically higher mPFS, ADCs demonstrate consistent and durable disease control, and chemo-immunotherapy shows more modest outcomes.** These comparisons are descriptive only and should not be interpreted as evidence of relative superiority due to differences in study design and patient populations.

Zongertinib is a preferred subsequent-line therapy for HER2-mutant NSCLC based on its targeted mechanism, strong activity demonstrated in clinical trials, favorable safety profile, and NCCN guideline endorsement. Zongertinib is an orally administered, irreversible TKI that selectively targets HER2, including exon 20 insertions, avoiding wild-type EGFR, helping to minimize target toxicities [11,14,52]. This selectivity is crucial, as it avoids severe skin rashes and diarrhea typically associated with pan-HER inhibitors that cross-react with EGFR. In the BEAMION LUNG-1 study, patients with HER2 TKD mutations who had received prior treatments experienced an ORR of 71% (Figure 3), a median duration of response of 14.1 months, and a mPFS of 12.4 months (Figure 4). Among those previously treated with both chemotherapy and HER2-directed ADCs, the ORR was 48%, with responses also seen in patients with brain metastases [14]. The ability of zongertinib to penetrate the blood–brain barrier addresses a significant unmet need, as CNS progression occurs in up to 30% of these patients. Safety was favorable, with low rates of grade ≥3 toxicities and no drug-related ILD [14]. This safety profile positions zongertinib as a versatile option for elderly patients or those with pre-existing pulmonary comorbidities. Recommended dosing is 120 mg daily for patients <90 kg and 180 mg daily for those ≥90 kg [53]. NCCN lists zongertinib as a preferred subsequent therapy for HER2-mutant NSCLC, noting that ranking does not imply preference [5]. Preclinical data show retained activity in trastuzumab deruxtecan-resistant models due to its distinct TKI mechanism [52] (Table 1).

On 26 February 2026, the U.S. FDA granted accelerated approval to zongertinib (Hernexeos; Boehringer Ingelheim Pharmaceuticals, Inc.), a kinase inhibitor, for the treatment of adults with unresectable or metastatic non-squamous NSCLC harboring HER2 (ERBB2) TKD activating mutations, as detected by an FDA-authorized test, who have not received prior systemic therapy [58].

### Sevabertinib: A Novel HER2 Tyrosine Kinase Inhibitor

Sevabertinib or “BAY 2927088” is an oral, reversible TKI that selectively targets tumors with EGFR/HER2 mutations, including exon 20 insertions, while sparing wild-type EGFR [55,59,60]. It was designed to overcome resistance mechanisms that limit the effectiveness of prior therapies. In practical terms, this selectivity is particularly relevant because it contributes to a more favorable therapeutic index, as earlier TKIs often showed off-target effects and dose-limiting toxicities. By targeting the mutant receptors, sevabertinib offers a superior therapeutic approach.

Clinical studies in patients with pretreated HER2-mutant NSCLC have shown that sevabertinib produces rapid, substantial, and durable responses. The ORR was 72.1%, the DCR was 83.7%, the median Duration of Response (DoR) was 8.7 months, and the mPFS was 7.5 months. In patients with HER2 YVMA insertions, the results were even higher: ORR was 90.0%, DCR was 96.7%, median DoR was 9.7 months, and mPFS was 9.9 months [19]. These findings highlight the potential of sevabertinib as a highly effective targeted therapy, particularly in molecularly selected populations where traditional therapies often yield limited benefit.

According to more current findings from the SOHO-01 trial, which was released in June 2025, the ORR for cohort D (81 patients) was 64%, for cohort E (55 patients) it was 38%, and for cohort F (73 patients) it was 71%. Diarrhea was the most common side effect of sevabertinib, though it was manageable. Notably, no cases of ILD or pneumonitis were reported (Table 1) [55]. From a clinical perspective, the absence of ILD is reassuring, especially when compared with other drugs in this class that have shown pulmonary toxicity to be a limiting factor.

Preclinical models confirm sevabertinib’s activity against ERBB2-encoded HER2 exon 20 insertions, including in cell lines resistant to first-generation TKI. Significantly, sevabertinib is a prospective therapy option for HER2-positive brain metastases since it can enter the central nervous system. Patients with baseline brain metastases in the SOHO-01 study had response rates of 61% (Cohort D), 27% (Cohort E), and 78% (Cohort F). For those without baseline CNS disease, only 5% (8 out of 167) had the central nervous system as the first site of progression [55]. This central nervous system activity is particularly meaningful and represents a major clinical advantage, as brain metastases remain a frequent and challenging complication in advanced NSCLC.

Ongoing clinical trials, including the SOHO-02 and panSOHO study, are evaluating sevabertinib’s safety, efficacy, and tolerability in solid tumors harboring HER2 or EGFR mutations. The SOHO-01 trial’s preliminary results point to a controllable toxicity and encouraging anticancer activity [61]. Additional data from these studies will help clarify its role and optimize its clinical use.

## 3. Novel Therapies for HER2 Mutations

### 3.1. SHR-A1811 (Trastuzumab Rezetecan): A Third-Generation HER2-Directed ADC

ADCs combine the specificity of monoclonal antibodies with the cytotoxic power of chemotherapy.

SHR-A1811 is a third-generation ADC composed of an anti–HER2 antibody named trastuzumab, and a unique topoisomerase I inhibitor payload SHR169265. This drug is described as a Potent, best-in-class anti-HER2 ADC with a highly permeable payload, optimized drug-to-antibody ratio, and better safety profiles [62,63]. This design allows efficient delivery of the cytotoxic agent directly to HER2-expressing tumor cells while minimizing systemic toxicity. The structural optimization of this ADC supports its intended function and contributes to its observed therapeutic activity.

Recent Phase 1/2 clinical trial results show that SHR-A1811 produces meaningful and durable responses in heavily pretreated HER2-mutant NSCLC, demonstrating consistent clinical activity across evaluated subjects. The ORR reached 38.1% overall and 41.9% at the recommended phase 2 dose (4.8 mg/kg), with a median DoR of 13.7 months and a DCR of 90.5% and mPFS of 9.5 months (Figure 4). Safety was acceptable, predominantly involving hematologic toxicities and ILD in 11.1% of individuals. ctDNA reductions correlated with tumor shrinkage and longer Progression Free Survival (PFS), supporting SHR-A1811 as a promising next-generation HER2-targeted ADC [56]. These results align with the expected pharmacological activity of the drug and support its continued evaluation.

SHR-A1811 is now being studied in phase II and III clinical trials across multiple tumor types, including breast, gastric, colorectal cancers, and NSCLC [63], indicating an ongoing effort to expand its therapeutic applicability across diverse HER2-expressing malignancies. These ongoing studies aim to further define its clinical benefit across different settings.

### 3.2. A166: A Potent HER2-Targeted ADC with Reduced Toxicity

A166 (also known as **trastuzumab botidotin**) is a next-generation HER2-targeted ADC consisting of a monoclonal antibody (trastuzumab) linked to the cytotoxic payload Duostatin-5 through a stable, protease-cleavable valine-citrulline linker [64]. This drug design enables selective intracellular delivery of its cytotoxic payload, reducing systemic exposure and enhancing tumor-specific activity while limiting the ocular and pulmonary toxicities seen with some ADCs. The emphasis on intracellular delivery mechanisms helps clarify the pharmacologic rationale behind minimizing off-target effects. From a practical standpoint, this design reflects growing awareness that drug delivery can be as important as target selection. This design approach reflects an effort to improve both tolerability and therapeutic precision compared to earlier ADCs.

Although A166 remains investigational for NSCLC, early safety and efficacy signals in HER2-positive lung cancer are being explored. In a phase I study involving patients with locally advanced or metastatic solid tumors, A166 has demonstrated good tolerability and encouraging antitumor activity in heavily pretreated HER2-positive malignancies. The ocular side effects observed were reversible and could be effectively managed with supportive care [65]. These early findings are consistent with more mature evidence now available in breast cancer, where similar safety observations have been described.

A recent Phase III trial involving 365 patients with HER2-positive metastatic breast cancer, A166 demonstrated superior efficacy compared to trastuzumab emtansine. The ORR was 76.9% versus 53.0%, and the mPFS was 11.1 months versus 4.4 months (hazard ratio 0.39, *p* < 0.0001). These advantages were consistent in prior lines of treatment. With a low rate of discontinuation due to adverse events (1.1%) and no treatment-related deaths reported, ocular toxicity remained controllable [64]. These results further reinforce the favorable balance between efficacy and safety observed with A166.

Overall, the available data support further investigation of A166 in HER2-driven cancers.

### 3.3. Zenocutuzumab: A Bispecific Antibody Targeting HER2 and HER3

Zenocutuzumab is a new, bispecific IgG1 antibody that blocks Neuregulin 1 (NRG1) binding and targets both HER2 and HER3 proteins. Oncogenic drivers known as NRG1 fusions have been found in solid tumors such as pancreatic ductal adenocarcinoma (PDAC) and NSCLC. *NRG1* fusions are rare, occurring in less than 1% solid tumors, and are known to drive resistance to HER2 monotherapies, which further contributes to the limited therapeutic options available for these patients [66]. Highlighting the clinical relevance of therapies specifically addressing this molecular alteration.

Zenocutuzumab became the first therapy to target *NRG1* following its accelerated approval by the FDA, marking an important regulatory milestone in precision oncology for rare genomic alterations. Under this approval, Zenocutuzumab is authorized for patients with pancreatic cancer or NSCLC with *NRG1* fusions whose disease has progressed despite standard therapies [67].

The approval was based on a phase II clinical trial involving 204 patients across 12 tumor types. Among 158 patients with measurable disease and ≥24 weeks of follow-up, the ORR was 30% (95% CI, 23–37), including 29% in NSCLC and 42% in pancreatic cancer. In total, 19% of responses were ongoing at the data cutoff, with a median response duration of 11.1 months. The mPFS was 6.8 months (95% CI, 5.5–9.1). Many adverse events were grade 1–2, with the most frequent ones being infusion-related responses (14%), nausea (11%), fatigue (12%), and diarrhea (18%), reflecting a generally manageable safety profile consistent with targeted antibody therapies and showing significant effectiveness in pancreatic cancer and NSCLC, with a preponderance of low-grade side effects [57].

## 4. Discussion

HER2 mutations represent a very small percentage of new patients diagnosed with NSCLC. However, due to the large number of new NSCLC cases seen every year, this represents thousands of patients annually who can benefit from this targeted therapy every year. Now with NGS available in the US in blood and tissue, these genomic alterations can now be identified more readily if we are looking for them at diagnosis or when patients develop resistance to front-line therapy that is something that provider and patient education can improve, because the rates of molecular testing in second line and beyond are very low, this has become increasingly clinically relevant with the availability of other options than chemotherapy and immunotherapy with three FDA-approved agents: trastuzumab deruxtecan (T-Dxd), zongertinib, and sevabertinib, and other agents in development. T-DXd was the first one used in second-line therapy, and we are waiting for randomized front-line data to see if it can be moved to the front line, but now we have serious competitors because being zongertinib and sevabertinib, oral agents that represent more attractive therapeutic approaches than endure prolonged intravenous (IV) therapy. Also, the toxicity profiles of the oral agents do not seem to be worse than T-DXd and certainly are more attractive than the option to give IV chemotherapy and immunotherapy. Moreover, the field is moving very rapidly, in February 2026, the FDA gave zongertinib first-line approval, moving this drug to the front line, and we are waiting for data with sevabertinib to probably do the same. The toxicity profile of all these agents allows them to be considered for the chronic therapy of these HER2 positive patients with manageable toxicity unless we see one of the few cases of ILD with T-Dxd. Future directions in HER2-altered NSCLC include the integration of targeted therapies into earlier lines of treatment, the development of rational sequencing strategies following resistance, and the refinement of biomarker selection through combined genomic and protein-based approaches. Ongoing clinical trials evaluating antibody–drug conjugates and next-generation tyrosine kinase inhibitors in the first-line setting are expected to further redefine treatment paradigms.

## 5. Conclusions

HER2 has historically been recognized as an important oncogenic driver in breast cancer, primarily through gene amplification and protein overexpression. More recently, activating HER2 mutations have emerged as clinically significant molecular alterations in NSCLC, contributing to tumorigenesis and representing actionable therapeutic targets.

The development of HER2-directed therapies, particularly ADCs and next-generation TKIs, has substantially expanded the therapeutic landscape and improved precision treatment strategies for patients with HER2-altered NSCLC. Consequently, comprehensive molecular characterization using NGS of both tissue and circulating tumor DNA should be strongly considered at diagnosis and at the time of disease progression or acquired resistance to facilitate appropriate patient selection and optimize treatment outcomes.

## Figures and Tables

**Table 1:** Efficacy and safety outcomes of therapies evaluated in HER2-mutant non-small cell lung cancer (NSCLC).

Therapy	ORR	DCR	mPFS	AE	Ref.
**Carboplatin+** **Pemetrexed+** **Pembrolizumab**	63.70%	83.50%	9.0 months	Asthenia; Anemia; Nausea	[54]
**Zongertinib**	71%	97%	12.4 months	Diarrhea; Rash; Nausea	[14]
**Sevabertinib** **(BAY 2927088)**	D: 64% E: 38% F: 71%	D: 81% E: 71% F: 89%	D: 8.3 months E: 5.5 months F:NR (Immature data)	Diarrhea; Nausea; Fatigue	[55]
**Trastuzumab rezetecan** **(SHR-A1811)**	38.10%	90.50%	9.5 months	Nausea; Decreased Neutrophil count; Anemia	[56]
**Zenocutuzumab**	30%	NR	6.8 months	Diarrhea; Musculoskeletal pain; Fatigue	[57]

**Note:** ORR, Disease Control Rate (DCR), and mPFS values and common Adverse Events(AE) summarized in this table are extracted from published studies and results are not intended for direct cross-trial comparison. Cohort-specific results for sevabertinib (BAY 2927088) are presented separately.

## Data Availability

No new data was created or analyzed in this study. Data sharing is not applicable.
